# F36. SELF-ESTEEM AND SYMPTOMS IN INDIVIDUALS AT CLINICAL HIGH-RISK FOR PSYCHOSIS

**DOI:** 10.1093/schbul/sby017.567

**Published:** 2018-04-01

**Authors:** Caridad Benavides, Gary Brucato, David Kimhy

**Affiliations:** 1 Icahn School of Medicine at Mount Sinai; 2 Columbia University

## Abstract

**Background:**

Individuals with psychotic symptoms often report low global self-esteem (GSE).

However, it remains unclear whether the low GSE is linked to the presence of psychotic symptoms or it is present prior to the onset of psychosis. Additionally, the specific subdomains of GSE in these populations are unknown.

**Methods:**

To address this question, we conducted a cross-sectional study comparing global and SE elements among individuals at clinical high-risk for psychosis (CHR; n=36), individuals with schizophrenia (SCZ; n=43), and healthy controls (HC; n=40). We then examined among CHR individuals the association between GSE, subdomains, and symptoms.

**Results:**

CHR individuals displayed significantly lower GSE compared to HC, at a level comparable to individuals with SCZ. The low GSE was driven primarily by self-perceptions of work and interpersonal relationships abilities, in the sub-domains of provider (r=.53, p<.001), physical appearance (r=.37, p=.026), sense of humor (r=.45, p=.006), intimate relations (r=.45, p=.006), and job competence (r=.39, p=.018). Lower GSE was associated with overall negative (p<0.01) and disorganized (p<0.05) symptoms severity, but not positive ones.

**Discussion:**

This investigation demonstrates that low self-esteem, which is prevalent in SCZ, is also present to a comparable degree of severity in CHR individuals. Our findings help to better understand the CHR period and may suggest targets for early psychosocial treatments aimed at improving social and occupational functioning, as such domains appear to play an important role in determining GSE in this population. The examination of different dimensions of self-esteem in the assessment of CHR populations may contribute to a better understanding of the phenomenology of prodromal symptoms, and thus, may have implications for finding successful early therapeutic approaches, rehabilitation, and social integration for a condition that is accompanied by considerable suffering and risk of suicide.

